# H1 Hemagglutinin Priming Provides Long-Lasting Heterosubtypic Immunity against H5N1 Challenge in the Mouse Model

**DOI:** 10.1128/mBio.02090-20

**Published:** 2020-12-15

**Authors:** Juan Manuel Carreño, Shirin Strohmeier, Ericka Kirkpatrick Roubidoux, Rong Hai, Peter Palese, Florian Krammer

**Affiliations:** a Department of Microbiology, Icahn School of Medicine at Mount Sinai, New York, New York, USA; b Department of Biotechnology, University of Natural Resources and Life Sciences, Vienna, Austria; c Graduate School of Biomedical Sciences, Icahn School of Medicine at Mount Sinai, New York, New York, USA; d Department of Microbiology and Plant Pathology, Institute for Integrative Genome Biology, University of California, Riverside, California, USA; e Department of Medicine, Division of Infectious Diseases, Icahn School of Medicine at Mount Sinai, New York, New York, USA; Johns Hopkins Bloomberg School of Public Health

**Keywords:** influenza, heterosubtypic immunity, stalk antibodies, imprinting

## Abstract

Influenza virus infections leave a signature of immune memory that influences future responses to infections with antigenically related strains. It has been hypothesized that the first exposure in life to H1N1 influenza virus imprints the host immune system, potentially resulting in protection from severe infection with H5N1 later in life through hemagglutinin (HA) stalk-specific antibodies. To study the specific role of the HA on protection against infection without interference of cellular immunity or humoral antineuraminidase immunity, we primed mice with influenza B viruses that express an H1 HA (group 1; B-H1), H3 HA (group 2; B-H3), or wild-type influenza B virus and subsequently challenged them at different time points with an H5N1 virus. Weight loss and survival monitoring showed that the B-H1-primed mice exhibited better protection against H5N1 compared to the control mice. Analysis of H5-specific serum IgG, before and 21 days after H5N1 challenge, evidenced the presence of anti-stalk H5 cross-reactive antibodies in the BH-1 group that were boosted by H5N1 infection. The increased immune responses and protection induced by priming with the B-H1 viruses lasted at least up to 1 year. Hence, a single HA priming based on natural infection induces long-lasting protective immunity against heterosubtypic strains from the same phylogenetic HA group in mice. This study gives mechanistic support to the earlier finding in humans that imprinting by H1 HA protects against H5N1 infections and that highly conserved regions on the HA, like the stalk, are involved in this phenomenon.

## Abstract (precis)

Current studies point out that an HA-mediated immunological imprint is established early in life during the first exposure to influenza viruses, which critically shapes and biases future immune responses. However, these findings have not been confirmed in animal models, and the precise mechanisms of this phenomenon are not clearly understood.

## INTRODUCTION

Influenza viruses are single-stranded, negative-sense, segmented RNA viruses belonging to the *Orthomyxoviridae* family. Four different types of influenza viruses (A, B, C, and D) are currently described, but only types A and B cause frequent seasonal outbreaks in humans. Influenza viruses encode at least 11 structural and nonstructural proteins, of which the external glycoproteins hemagglutinin (HA) and neuraminidase (NA) constitute the major antigenic targets (1, 2). Indeed, influenza viruses can be classified based on the antigenicity of the glycoproteins on their surface. Among influenza A viruses, different subtypes of HAs (18 subtypes) and NAs (11 subtypes) have been described (3). Likewise, HAs are subclassified into group 1 (H1, H2, H5, H6, H8, H9, H11, H12, H13, H16, H17, and H18) and group 2 (H3, H4, H7, H10, H14, and H15) subtypes based on the phylogeny of the HA gene. In particular, HA has been antigenically dissected, and the major antigenic determinants of different HA subtypes have been identified (4–9).

Immunodominance is partially based on properties intrinsic to the antigen (10). Defined patterns of HA immunodominance upon exposure to a particular influenza virus strain/subtype can be identified in human subjects and animal models (9). Large differences between the head and stalk domains of the HA are appreciated (11). The head of the HA, which consists of an exposed globular domain located on the top of the glycoprotein, is highly immunodominant. The stalk domain, which is membrane proximal, is immunosubdominant and is highly conserved among strains from the same phylogenetic group (12–16). HA immunodominance patterns of different strains have been characterized. For instance, H1N1 viruses, which carry a group 1 HA, display five major antigenic sites that surround the receptor binding site (RBS): Sa, Sb, Ca1, Ca2, and Cb (5, 6). H3N2 also displays five major antigenic sites in the head domain (A, B, C, D, and E); however, their structure and distribution are substantially different (4, 7, 8). Hence, differences in the sequence and structure of the HAs from different strains/subtypes contribute to the particular patterns of immunodominance in humans and animal models (9, 11, 17).

However, immunodominance is further complicated by the immune history of a particular host. Extensive evidence suggests that the immune responses, and particularly the antibody responses induced in humans during an influenza virus infection, are shaped by previous encounters to influenza viruses and exposure to vaccine antigens (11, 18, 19). Further exposures to heterosubtypic strains might increase the breadth of antibodies toward conserved epitopes between subtypes from the same phylogenetic group (13), from a distinct phylogenetic group (14), or even toward a different type of influenza virus (15). Immunization studies with H5N1 (group 1) and H7N9 (group 2) vaccines show that the immune responses elicited after exposure to these phylogenetically distant subtypes are mostly directed toward conserved regions between the new strain and the previous strains that an individual has encountered (20–22). Interestingly, individuals infected early in life with H1N1 (group 1) have been hypothesized to be better protected from severe morbidity and mortality caused by zoonotic H5N1 (group 1), while individuals initially exposed to H3N2 (group 2) may exhibit a similar protection when exposed to zoonotic H7N9 (group 2) (23, 44). Cross-group imprinting has recently also been described in animal models and humans, but its relevance so far is unclear (45, 46). Importantly, these effects have been mostly attributed to the HA, but the specific HA-based protection induced by natural infection with particular strains—sharing many epitopes on different proteins—is difficult to test in humans. Therefore, we designed a model to study this phenomenon *in vivo*. For this, we primed mice with influenza B viruses that express the HA from either group 1 or group 2 influenza A viruses (B-HA influenza viruses) and then assessed protection against H5N1 virus challenge at different time points throughout the life span of the mice. Our results indicate that a single HA-based priming is enough to induce long-lasting protective immunity against heterosubtypic strains from the same phylogenetic group.

## RESULTS

### Model to study the effect of HA-specific immunity on protection against H5N1 infection in mice.

**FIG 1:**
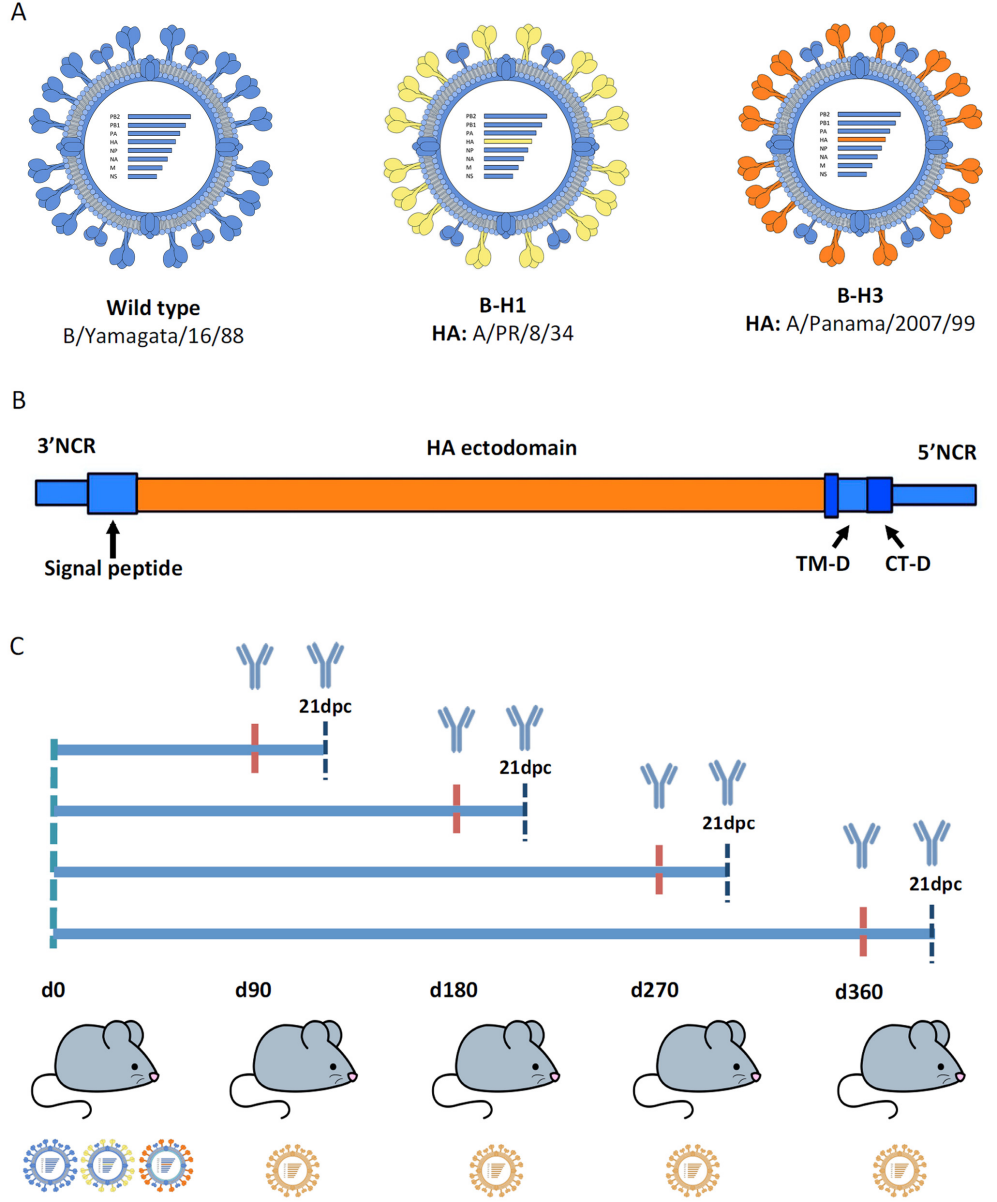
Generation of a model to study HA-specific heterosubtypic immunity in mice. (A) Recombinant B/Yamagata/16/88 viruses (B-HA viruses) expressing either wild-type B/Yamagata/16/88 HA or H1 (group 1) from A/PR/8/34 (B-H1; depicted in yellow) or H3 (group 2) from A/Panama/2007/99 (B-H3; depicted in orange). The genomic segments for expression of H1 and H3 via recombinant influenza B viruses include the packaging signals, transmembrane (TM-D) and endodomain (CT-D) derived from the influenza B virus HA (all depicted in blue) and the ectodomain from influenza A virus (depicted in orange). NCR, noncoding region. (B) Diagram of the study. Mice were sublethally infected with B-H1, B-H3, or wild-type influenza B virus, and at 90, 180, 270 or 360 days postpriming, mice were challenged with A/Vietnam/1203/04 H5N1 virus (PR8 reassortant, polybasic cleavage site deleted) at doses ranging from 10^1^ to 10^5^ PFU/mouse. (C) Weight loss and survival kinetics were measured following H5N1 challenge. Antibody responses, including effector functions, were measured prechallenge and 21 days postchallenge (dpc).

First exposures in life to influenza viruses are crucial for shaping future immune responses against heterologous (same subtype but different strain) strains and potentially against heterosubtypic strains. Humans whose primary exposure was to group 1 influenza viruses such as H1N1 are expected to have a better outcome against infections with heterosubtypic viruses from the same phylogenetic group such as H5N1 (23, 44). This group-specific protection has been associated with immune responses toward regions conserved between group 1 influenza viruses (20, 22, 23, 44). However, it is complicated to eliminate the influence of other factors and assess only the HA contribution in protection during a natural infection in humans. Moreover, the duration of these particular cross-protective responses has not been fully assessed in animal models. Here we studied the specific role of the HA from distinct phylogenetic groups on cross-protection against infection with the heterosubtypic strain H5N1. For this purpose, we used a model based on influenza B viruses that express HAs from different influenza A viruses (B-HA viruses) (Fig. 1A). The backbone of these viruses is based on B/Yamagata/16/88 virus expressing either H1 (group 1; B-H1) from A/PR/8/34 or H3 (group 2; B-H3) from A/Panama/2007/99 (24). The HA genomic segment of these viruses is constructed to have the packaging signals, transmembrane, and endodomain from influenza B virus but the ectodomain from influenza A virus (Fig. 1B) (24). The use of these viruses allows us to assess the contribution of HA-specific immunity in protection against a heterosubtypic strain without the interference of cellular immunity to the conserved internal proteins or humoral antineuraminidase immunity to influenza A virus.

For priming infections, groups of mice (*n* = 60/group) were initially infected with (i) B-H1, (ii) B-H3, or (iii) influenza B virus (wild type). An amount of virus necessary for production of high antibody titers with comparable HA-specific antibody levels among the different groups was used (B-H1, 5 × 10^2^; B-H3, 1 × 10^6^; and wild type, 5 × 10^5^ PFU/mouse, respectively). A control group of mice (*n* = 60) received phosphate-buffered saline (PBS) only. At 90, 180, 270, or 360 days postpriming, mice from each group were challenged with A/Vietnam/1203/04 H5N1 (PR8 reassortant, as described above) virus using five different doses (10^1^, 10^2^, 10^3^, 10^4^, or 10^5^ PFU/mouse, *n* = 3 mice per dose). Weight loss kinetics and survival after every challenge were monitored. Antibody responses were determined prior to every challenge and 21 days after the challenge. Effector functions of antibodies were assessed 21 days after challenge as well. Every panel of assays was repeated consistently for each of the four different time points (Fig. 1C).

### B-H1 priming provides long-lasting protection against H5N1 challenge.

**FIG 2:**
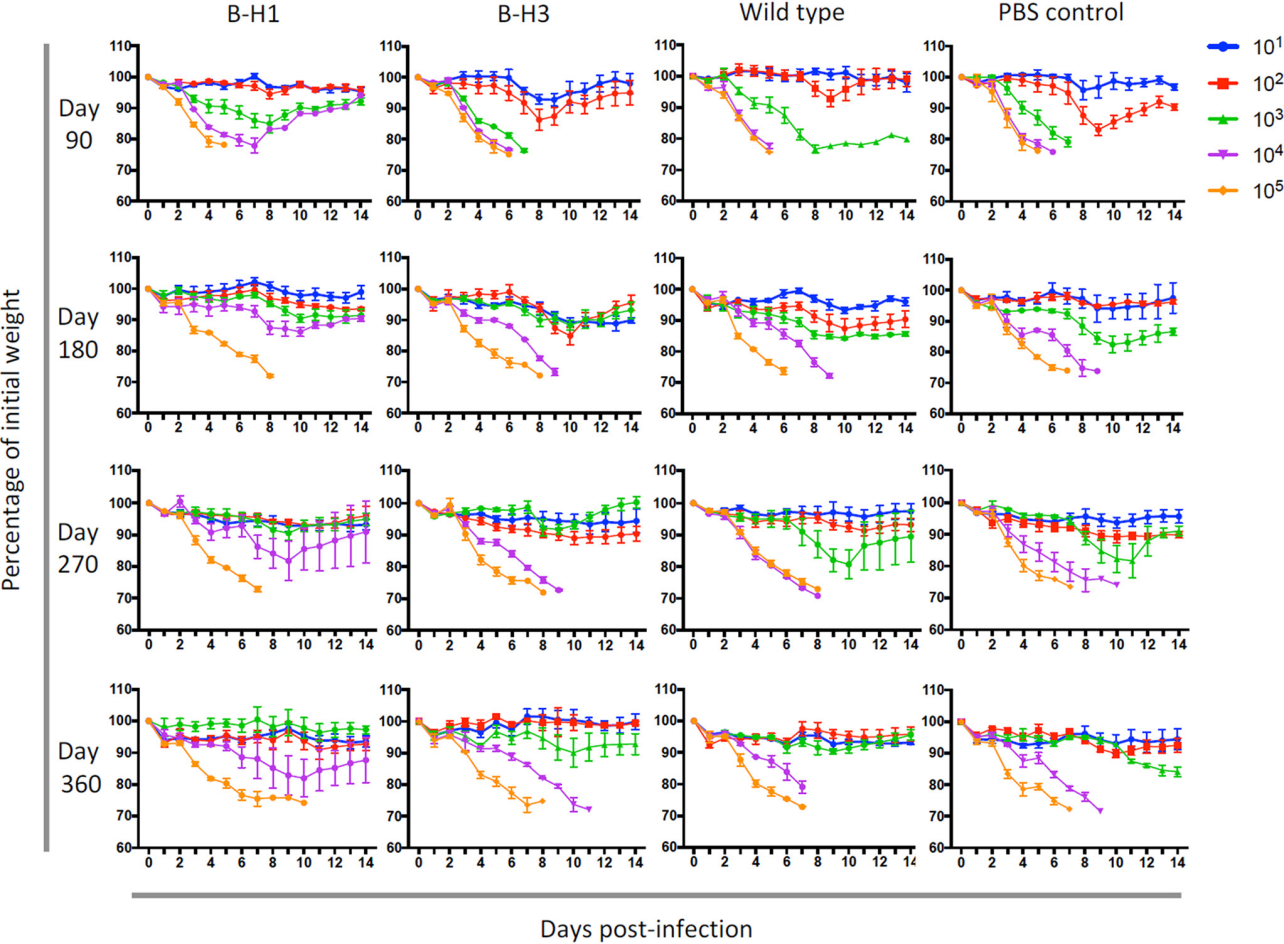
B-H1 priming reduces mouse weight loss during H5N1 challenge. Mice were primed with B-H1, B-H3, or B/Yamagata/16/88 (wild-type) virus or administered PBS (*n* = 60/group). At 90, 180, 270, or 360 days after priming, mice were infected with H5N1 A/Vietnam/1203/04 (PR8 reassortant) virus at five different doses (10^1^, 10^2^, 10^3^, 10^4^, or 10^5^ PFU/mouse, *n* = 3 mice per dose). Weight monitoring was performed daily for 14 days. Weight is expressed as the percentage of initial weight at day 0 (100%). The mean ± standard error of the mean (SEM) (error bar) is plotted for each group.

**FIG 3:**
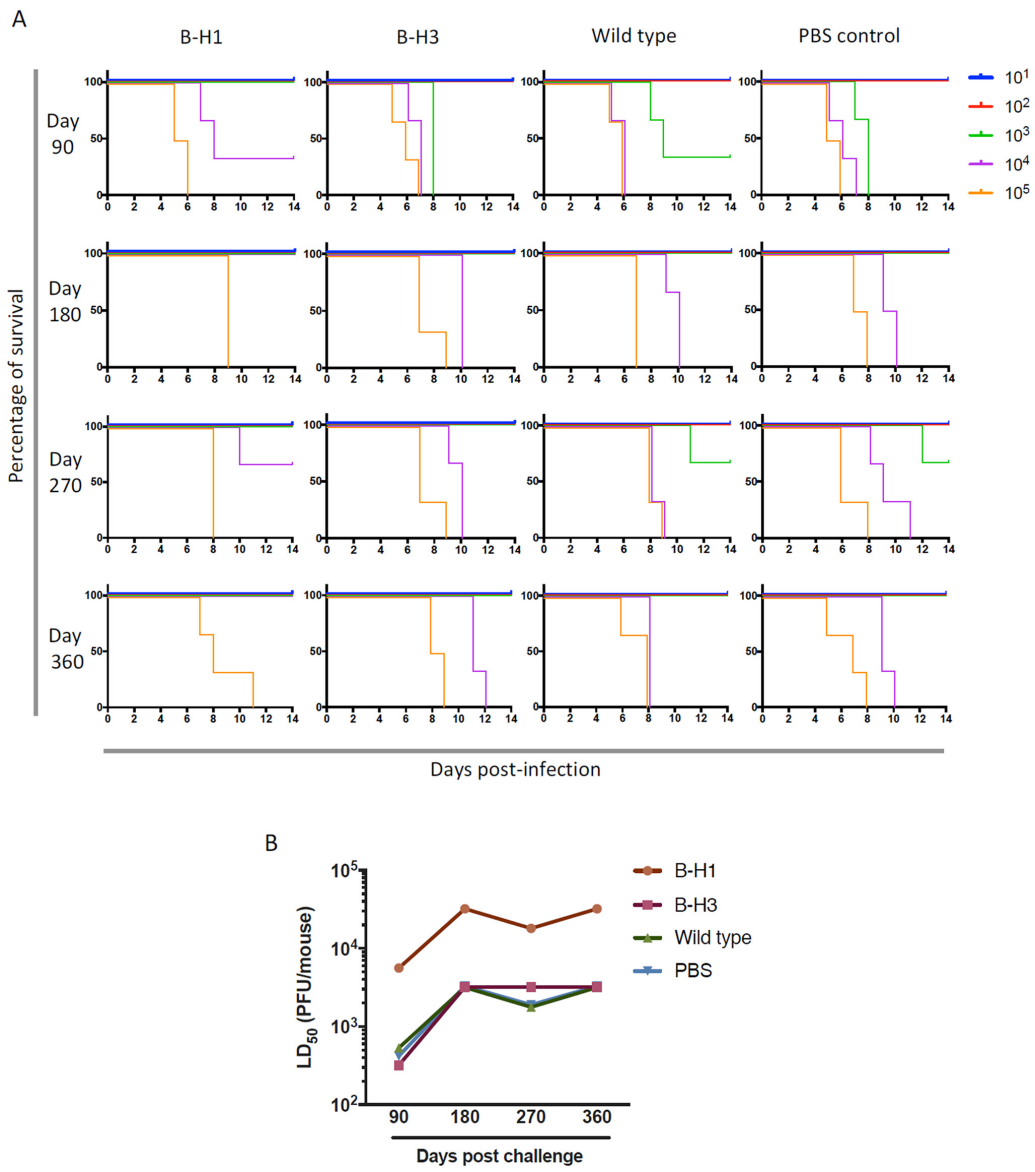
B-H1 priming increases mouse survival following H5N1 challenge. Mice were primed with B-H1, B-H3, or B/Yamagata/16/88 (wild-type) virus or administered PBS (*n* = 60/group). At 90, 180, 270, or 360 days after priming, mice were infected with H5N1 A/Vietnam/1203/04 (PR8 reassortant) virus at five different doses (10^1^, 10^2^, 10^3^, 10^4^, or 10^5^ PFU/mouse, *n* = 3 mice per dose). (A) Survival monitoring was performed daily for 14 days. Mice were euthanized when weight loss exceeded 25% of initial weight. (B) The 50% lethal dose (LD_50_) for every group of primed mice at every time point was calculated. Every symbol in the graph represents a single LD_50_ value.

Heterosubtypic immune responses induced by vaccination with H5N1 have been assessed (20, 22). However, studies of heterosubtypic protection *in vivo*, induced by natural infection, and in the context of HA-specific responses are limited. Therefore, we analyzed the role of HA-specific immune responses on protection of mice initially primed with B-H1, B-H3, and influenza B virus against challenge with A/Vietnam/1203/04 H5N1 virus. For measurements of disease progression in mice, we assessed weight loss kinetics and survival during 14 days after the challenge. Only mice that were initially primed with B-H1 viruses exhibited enhanced protection against challenge with H5N1 virus. Mice from the B-H1-primed group not only had a reduction in weight loss when they received 10^1^ to 10^3^ PFU/mouse of H5N1 virus at different time points (Fig. 2), but mice receiving 10^3^ PFU/mouse survived at day 90 and most of the mice receiving 10^4^ PFU/mouse survived at all time points (Fig. 3A). In contrast, mice initially primed with viruses containing a group 2 HA (B-H3) or an influenza B virus HA or that received PBS displayed higher morbidity and lower survival at all time points. Importantly, analysis of the overall 50% lethal dose (LD_50_) in the different groups at every time point confirms that the protection induced by the B-H1 priming is preserved over time, and even though the LD_50_ is lower at 90 days postinfection, the overall pattern of protection per time point is similar (Fig. 3B; see also Table S1 in the supplemental material). On average, H1 priming increased resistance to H5N1 challenge 10-fold over the control groups at all time points. These results may support findings about the protective effect of H1N1 imprinting against H5N1 infection in humans (23, 44).

10.1128/mBio.02090-20.1 TABLE S1 Differential lethality of H5N1 in mice primed with heterosubtypic strains. Download Table S1, PDF file, 0.1 MB. Copyright © 2020 Carreño et al. 2020 Carreño et al. This content is distributed under the terms of the Creative Commons Attribution 4.0 International license.

### Immune responses to conserved regions of group 1 HAs mediate protection in B-H1-primed mice.

**FIG 4:**
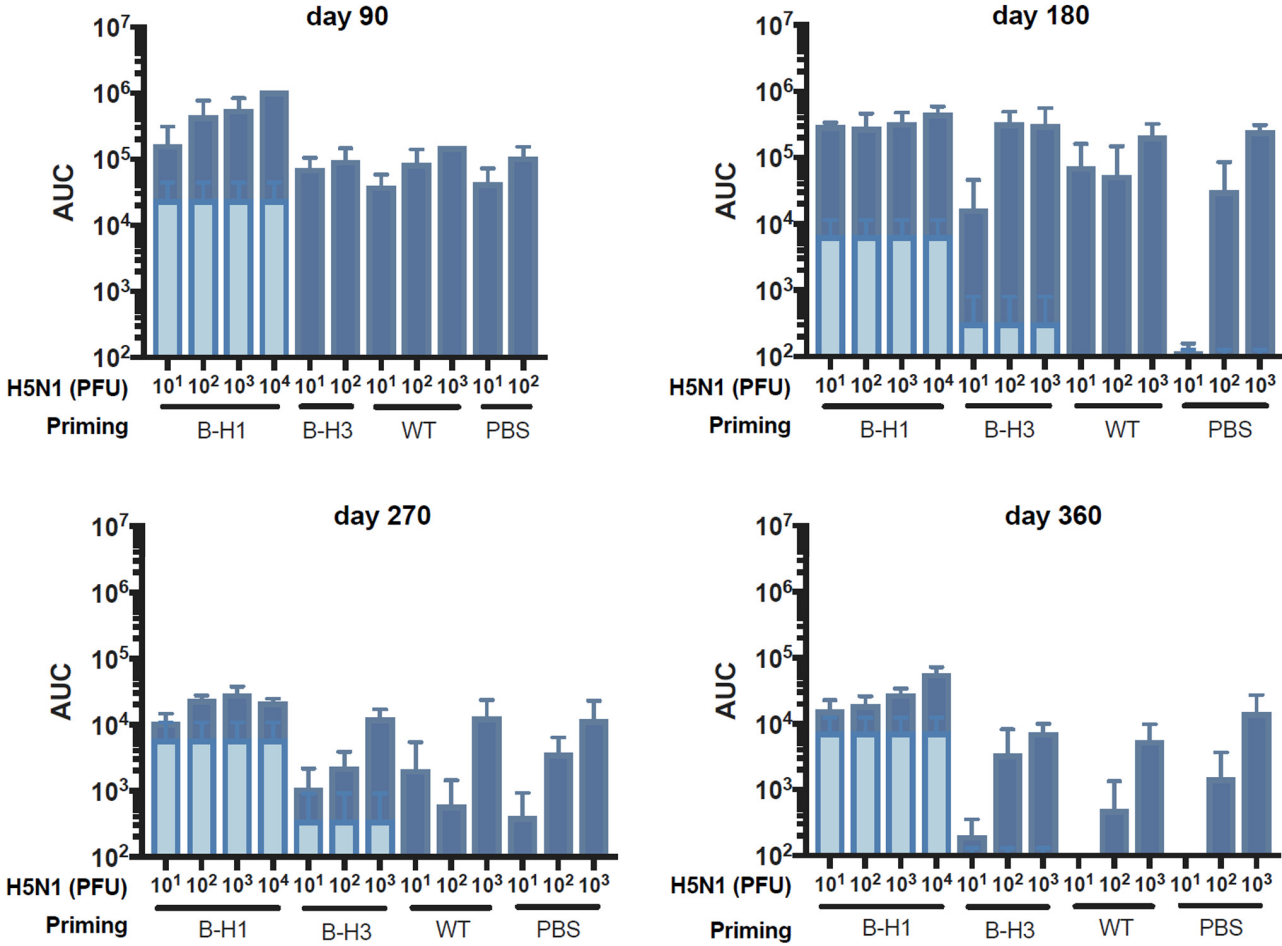
Increased H5-specific antibodies in B-H1 primed mice pre- and post-H5N1 challenge. Mice were primed with B-H1, B-H3, or B/Yamagata/16/88 virus (wild type [WT]) or treated with PBS (*n* = 60/group). At 90, 180, 270, or 360 days postpriming, sera from all the mice were collected. Mice were challenged with 10^1^, 10^2^, 10^3^, 10^4^, or 10^5^ PFU/mouse of H5N1 A/Vietnam/1203/04 (PR8 reassortant) virus (*n* = 3 mice per dose). At 21 days postchallenge, sera were collected from the mice that survived the infection. IgG against recombinant H5 from A/Vietnam/1203/04 was measured in pre- and postchallenge sera. Prechallenge H5-reactive IgG levels are shown as an average of all the primed mice from a single group at a specific time point (light blue bars). Postchallenge H5-reactive IgG levels are shown as an average of the primed mice from a single group at a specific time point and receiving the indicated dose of H5N1 virus (dark blue bars). Antibody levels are expressed as area under the curve (AUC). The mean plus SEM is plotted for each group.

Protection induced after influenza virus infection can be mediated either by specific antibodies typically directed against the surface glycoproteins of the virus (25) or by specific T cell responses (26). Here, we focused on the specific antibody responses induced by natural infection toward the HA. We analyzed the antibody responses in the mice primed with B-H1, B-H3, and influenza B virus at all time points (day 90, 180, 270, or 360) and either before the challenge with A/Vietnam/1203/04 H5N1 virus or 21 days after challenge. Measurement of specific IgG levels against recombinant H5 from A/Vietnam/1203/04 by enzyme-linked immunosorbent assay (ELISA) indicates that prechallenge cross-reactive antibodies against H5 are higher in mice primed with B-H1 viruses at all time points and that these levels of antibodies remain stable over time, up to day 360 (Fig. 4, light-colored bars). Moreover, these H5-reactive antibodies in the B-H1-primed groups were boosted by H5N1 challenge to reach very high levels with area under the curve (AUC) values up to 10^6^, and the increase in antibody levels being dose dependent (Fig. 4).

**FIG 5:**
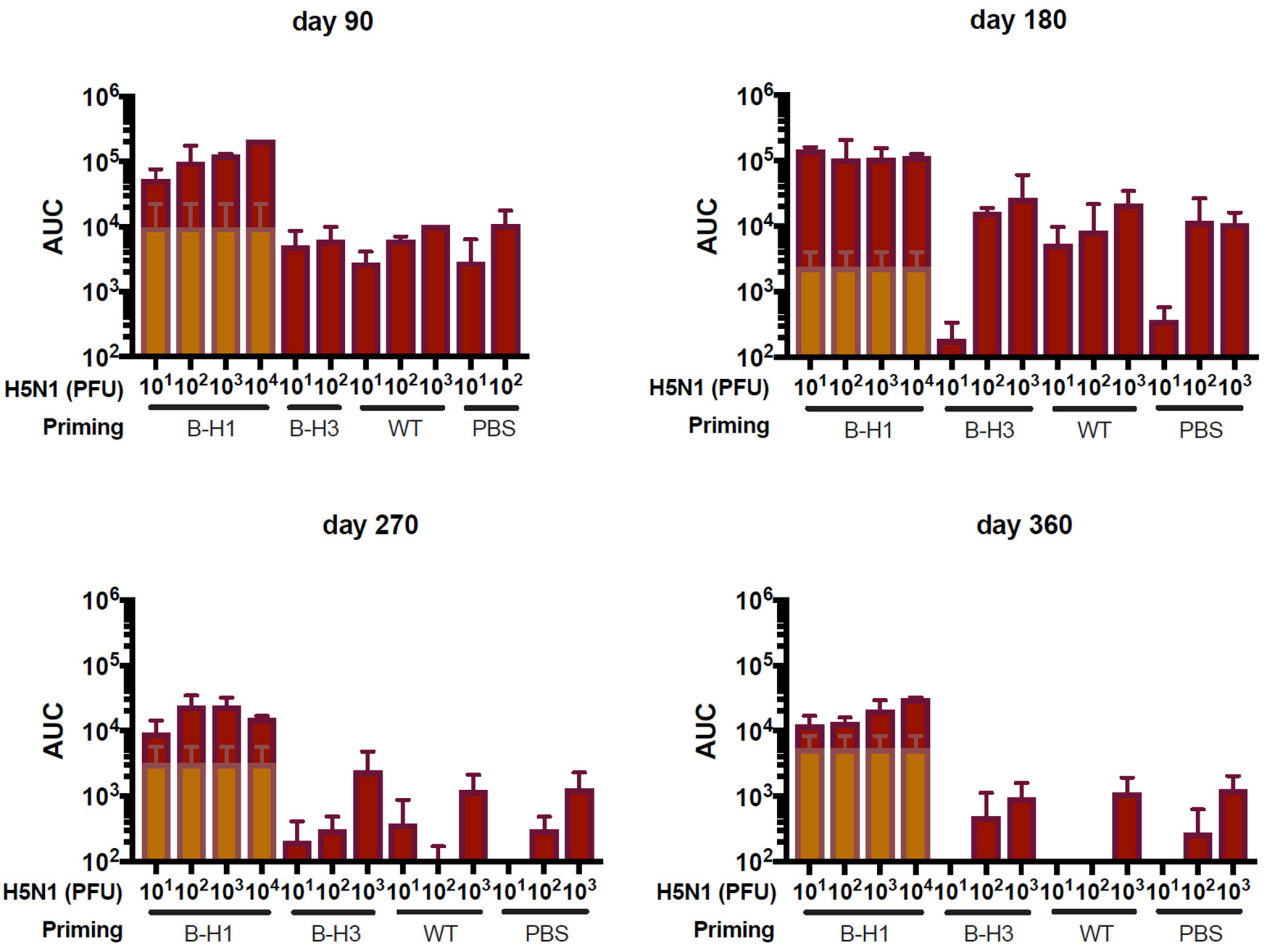
Increased stalk-specific antibodies in B-H1-primed mice pre- and post-H5N1 challenge. Mice were primed with B-H1, B-H3, or B/Yamagata/16/88 (wild-type [WT]) virus or treated with PBS (*n* = 60/group). At 90, 180, 270, or 360 days postpriming, sera from all the mice were collected. Mice were challenged with 10^1^, 10^2^, 10^3^, 10^4^, or 10^5^ PFU/mouse of H5N1 A/Vietnam/1203/04 virus (*n* = 3 mice per dose). At 21 days postchallenge, sera were collected from the mice that survived the infection. Specific IgG against the chimeric HA cH6/1, was measured in pre- and postchallenge sera. Prechallenge stalk-reactive IgG levels are shown as an average of all the primed mice from a single group at a specific time point (light brown bars). Postchallenge stalk-reactive IgG levels are shown as an average of the primed mice from a single group at a specific time point and receiving the indicated dose of H5N1 virus (dark brown bars). Antibody levels are expressed as area under the curve (AUC). The mean plus SEM is plotted for each group.

Next, we analyzed the specific antibody responses against the stalk of the HA by using a chimeric HA (cH6/1)-based ELISA (22). Prechallenge antibody levels were induced in mice primed with B-H1 viruses, and these levels of stalk-specific antibodies remained stable over time up to day 360 (Fig. 5, light-colored bars). Furthermore, antibody titers in the B-H1-primed groups were boosted by H5N1 challenge and reached high levels with AUC values above 10^5^. Moreover, the increase in antibodies was virus dose dependent (Fig. 5). No drastic changes were observed in prechallenge IgG levels against the specific HA from the corresponding priming strains (H1 for B-H1, H3 for B-H3, and B-HA for influenza B virus) at 90, 180, 270, or 360 days after the priming (see Fig. S1 in the supplemental material). Overall, these results indicate that priming with B-H1 viruses induced antibody responses against H1 that cross-react with H5 HA and that these antibodies are mostly directed toward conserved regions on the HA stalk.

10.1128/mBio.02090-20.2 FIG S1 Prechallenge IgG levels against the specific HA from the corresponding priming strains, H5 and cH6/1. Mice were primed with B-H1, B-H3, or B/Yamagata/16/88 virus or treated with PBS (*n* = 60/group). At 90, 180, 270, or 360 days postpriming, sera from all the mice were collected. Specific IgG against the corresponding HA from the priming strains (H1 for B-H1, H3 for B-H3, and B-HA for B-WT) (A), the chimeric HA cH6/1 (B), or H5 from A/Vietnam/1203/04 virus (C) was measured. Antibody levels are expressed as area under the curve (AUC). The mean plus SEM is plotted for each group. Download FIG S1, PDF file, 0.1 MB. Copyright © 2020 Carreño et al. 2020 Carreño et al. This content is distributed under the terms of the Creative Commons Attribution 4.0 International license.

### Antibodies induced by H5N1 challenge exhibit effector functions.

**FIG 6:**
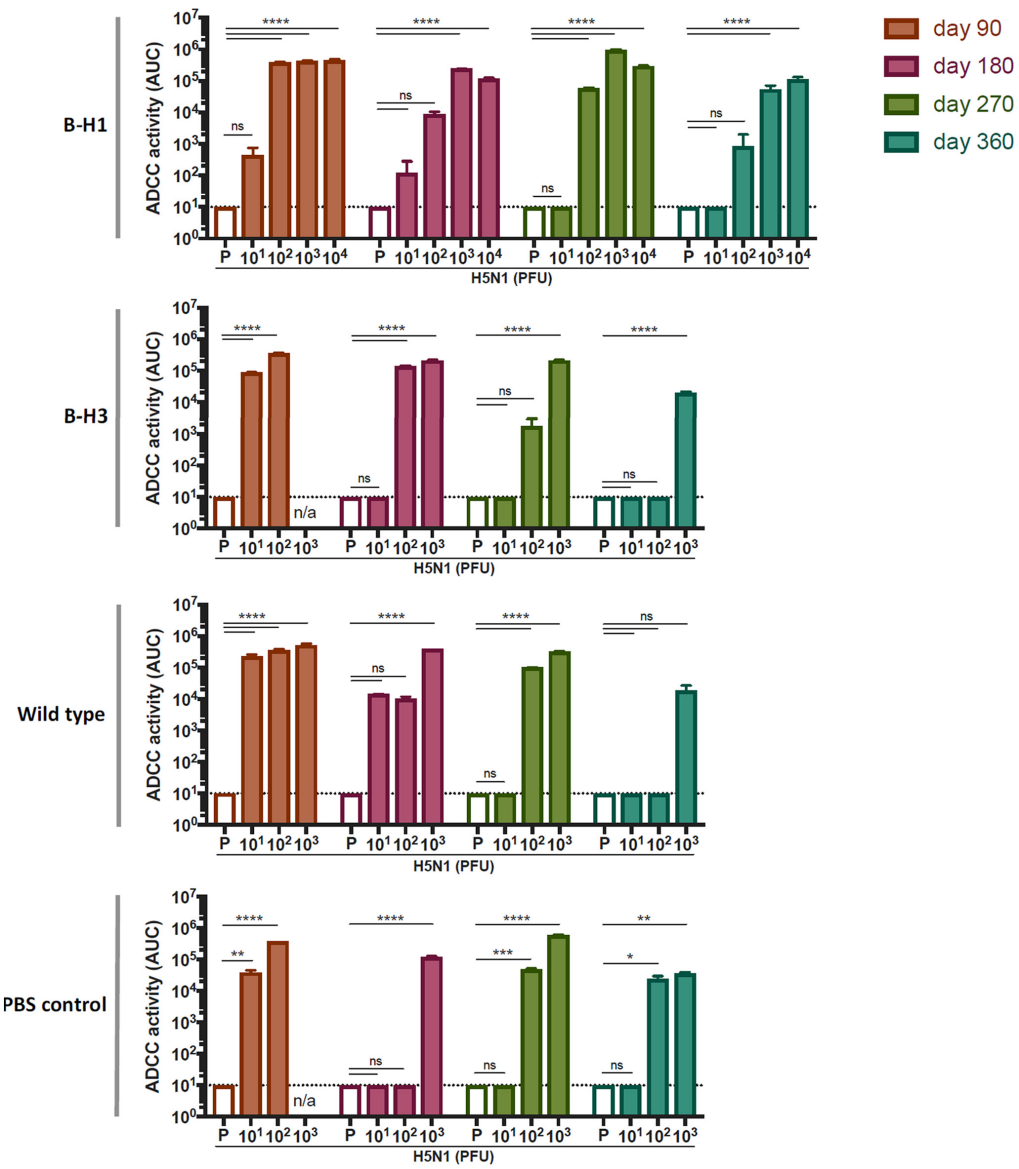
Induction of antibodies with ADCC activity following H5N1 challenge. Mice were primed with B-H1, B-H3, or B/Yamagata/16/88 (wild-type) virus or treated with PBS (*n* = 60/group). At 90, 180, 270, or 360 days postpriming, sera from all the mice were collected to determine prechallenge ADCC activity (empty bars). Mice were challenged with 10^1^, 10^2^, 10^3^, 10^4^, or 10^5^ PFU/mouse of H5N1 A/Vietnam/1203/04 virus (*n* = 3 mice per dose). At 21 days postchallenge, sera from all the mice were collected. Postchallenge ADCC activity was determined from pooled serum samples from a single group at a specific time point and receiving the indicated dose of H5N1 virus (colored bars). An average of technical replicates of pooled sera is shown. ADCC activity is expressed as area under the curve (AUC). The mean plus SEM is plotted for each group. Statistical significance is indicated as follows: ns, not significant; *, *P* < 0.0454; **, *P* < 0.0037; ***, *P* < 0.0007; ****, *P* < 0.0001. n/a, not available (mice did not survive the challenge).

Antibodies induced after influenza virus infection may display effector functions that contribute to virus clearance and recovery from disease (27). In particular, HA stalk-specific antibodies have an intrinsic capacity to activate these cellular mechanisms, such as antibody-dependent cellular cytotoxicity (ADCC) (27, 28). To assess the effector functions of antibodies induced after H5N1 challenge in mice that were primed with B-H1, B-H3, influenza B virus, or administered PBS, we used a commercial reporter assay kit to measure the ADCC activity in serum. Samples from each group (B-H1, B-H3, influenza B virus, or PBS), at every time point (day 90, 180, 270, or 360) were pooled according to the dose of H5N1 virus received (*n* = 3 mice per group). Most of the mice receiving above 10^2^ PFU/mouse of H5N1 displayed antibodies with strong ADCC reporter activity after challenge (Fig. 6), and this effect was maintained over time in aging mice. Of note, prechallenge sera from mice that received only priming did not display measurable ADCC activity in any of the groups at any time point (Fig. 6), suggesting that the presence of H5 cross-reactive antibodies with effector functions prior to the challenge with H5N1 is limited but might rapidly be boosted after challenge.

## DISCUSSION

Immunodominance and preexisting immunity are two major elements that modulate influenza virus infections (11, 18, 19). Here, we used a model based on influenza viruses bearing HAs from distinct phylogenetic groups in a homologous genetic background. Hence, we were able to study the specific contribution of HA in the induction of protective immunity against a group 1 HA heterosubtypic strain. By priming mice with the B-HA viruses, we could avoid cellular immunity to the internal proteins or humoral anti-NA immunity, allowing us to study the impact of HA-specific immunity only. Moreover, the relatively short life span of mice allowed us to evaluate the duration of priming-induced protection at different stages of aging.

Immunosenescence is characterized by deterioration of the immune system, lower responses to vaccination, and an overall decline in antibody and cellular immune responses (29). Here, we found that antibody responses following H5N1 challenge decreased over time in all the groups. However, stalk antibodies induced in young mice did not wane over time and still offered protection against H5N1 challenge in aging mice. In general, older mice in all groups became more resistant to H5N1 challenge over time. The higher resistance of older mice to virus infection in general is in agreement with previous studies describing reduced influenza virus pathogenicity in aging mice (30), likely due to reduced local tissue inflammation and immunopathology (30, 31). Moreover, while altered inflammatory responses are potentially responsible for the resistance to H5N1 challenge in aged mice, it is also possible that the increased weight of older mice reduced mortality (since the humane endpoint is based on weight loss). Importantly, priming with B-H1 viruses induced long-lasting heterosubtypic protection against H5N1 that was sustained until day 360 and provided approximately 10-fold-higher resistance to challenge compared to the other groups of mice.

Our results indicate that priming with B-H1 viruses induced antibody responses against H1 that are cross-reactive to H5. Importantly, these antibodies are mainly directed toward conserved regions of the stalk domain. The amino acid sequence identity between different H1 HAs and H5 from H5N1 A/Vietnam/1203/04 virus (the strain used in this study) oscillates around 65% for the full-length HA, 46 to 50% for the head domain, and importantly, 77 to 78% identity for the stalk domain (32). Antibody responses toward conserved regions of the stalk domain have a demonstrated protective potential in both preclinical models (14, 15, 26) and in humans (33–36). Therefore, the cross-reactive responses seen here are likely to be involved in the improved clinical features and increased survival in the B-H1-primed groups. Furthermore, these stalk-specific antibodies were boosted upon H5N1 challenge, perhaps through a secondary affinity maturation process, increasing the breadth of the stalk-reactive repertoire, as has been reported for antibodies directed toward the RBS (37). Although we did not assess the complex scenarios of multiple infections occurring in humans, our model could eventually be employed to study the specific role of HA on the mechanisms of imprinting. In terms of the effector functions of the antibodies, we detected a dose-dependent induction of ADCC reporter activity followed by H5N1 challenge, regardless of the priming strain. This would suggest that the responses observed are likely due to the magnitude of H5N1 replication in the different groups. It has been shown that an increase in antibodies with ADCC reporter activity following pandemic H1N1 infection correlates with preexisting cross-reactive immunity (38). It is very interesting that in our study we did not detect prechallenge ADCC activity against H5N1 in any of the groups, despite detecting H5 cross-reactive antibodies. Whether this effect is due to the limit of detection of the assay or other reasons remains to be further explored.

For future studies, it would also be interesting to assess the contribution of T cells in the enhanced protection seen in B-H1-primed mice. Cross-reactive T cells, particularly CD8^+^ cells, are able to recognize epitopes from influenza A viruses of different subtypes (47), from influenza B viruses of distinct lineages (48), and from seasonal and pandemic influenza viruses (47, 49, 50) and even display cross-reactivity among influenza A, B, and C viruses (51). However, few cross-reactive CD8^+^ T cells are typically found against conserved epitopes in the HA, and rather, these responses are directed toward internal proteins of the virus (49–51). Cross-reactive CD4^+^ T cells have been described as well (49, 52, 53). Interestingly, preexisting cross-reactive CD4^+^ T cells have been associated with lower virus shedding and less severe illness in humans (52). Moreover, CD4^+^ T cells are able to target cross-reactive epitopes in the HA (53). Hence, it would be of interest to test if B-H1-primed mice elicited HA cross-reactive CD8^+^ or CD4^+^ T cells protecting against H5N1 infection.

Our study underlines the importance of a proper “education” of the immune system during early stages in life. Using the right stimuli by means of strategic vaccination regimens and understanding the mechanisms that shape commitment of immune responses to certain antigens are crucial. Reduced vaccine effectiveness for seasonal influenza is mainly due to mismatched strains (39). Even when the vaccine HA is a good match, vaccine failures occur due to inadequate host immune responses, influenced by an individual’s early-life influenza exposure history (18, 40, 41). Therefore, implementation of vaccines with a wide breadth of protection, such as universal influenza virus vaccines (42), particularly early during childhood (43), might have a substantial impact. In summary, we designed an *in vivo* mouse model to study protective, cross-reactive antibody responses specific for the HA, induced during natural infection. Importantly, a single priming with H1 HA induced long-lasting resistance against a heterosubtypic virus from the same phylogenetic group. Our model might prove useful for future studies assessing HA-specific priming/imprinting responses in different complex scenarios of infection and vaccination.

## MATERIALS AND METHODS

### Cells, viruses, and proteins.

Cells used for ADCC reporter assays: Madin-Darby canine kidney (MDCK) cells were cultured in Dulbecco’s modified Eagle’s medium (DMEM) supplemented with 10% fetal bovine serum (FBS; Sigma-Aldrich) and penicillin (100 U/ml)-streptomycin (100 μg/ml) solution (Gibco). ADCC Jurkat effector cells expressing mouse Fcγ receptor IV (FcγRIV) were grown in Roswell Park Memorial Institute (RPMI) 1640 medium supplemented with l-glutamine (Gibco) and 10% fetal bovine serum (HyClone), 100 μg/ml hygromycin (Invitrogen), 250 μg/ml antibiotic G-418 sulfate solution (Sigma-Aldrich), 1 mM sodium pyruvate (Gibco), and 0.1 mM minimal essential medium (MEM) nonessential amino acids (Gibco). Viruses used for priming-infection had a backbone of B/Yamagata/16/88 and expressed either H1 (group 1; B-H1) from A/PR/8/34 or H3 (group 2; B-H3) from A/Panama/2007/99. Wild-type B/Yamagata/16/88 virus (influenza B virus) was used as a control. For challenge experiments, an H5N1 virus (A/Vietnam/1203/04, 6:2 reassortant with an A/PR/8/34 backbone and polybasic cleavage site deleted) was used. Viruses were grown in 8-day-old embryonated eggs (Charles River Laboratories) at 37°C for 48 h (challenge virus) and 33°C for 72 h (priming viruses). For enzyme-linked immunosorbent assays (ELISAs), recombinant proteins expressed in the baculovirus system were used: cH6/1 (described above) to assess stalk-specific antibody responses, H1 from H1N1 virus (A/PR/8/34), H3 from H3N2 virus (A/Hong Kong/4801/2014), HA from B/Yamagata/16/88, and H5 from H5N1 virus (A/Vietnam/1203/04).

### Mouse infections.

All experiments with mice were performed in accordance to protocols approved by the Icahn School of Medicine at Mount Sinai Institutional Animal Care and Use Committee. Blood samples were obtained by submandibular bleeding. To perform infections, 6- to 8-week-old mice were anesthetized using 0.1 ml of 0.15 mg of ketamine/kg of body weight and 0.03 mg of xylazine/kg intraperitoneally. Mice were initially infected intranasally with 50 μl of a PBS solution containing an amount of B-H1, B-H3, and wild-type influenza B priming viruses necessary to yield high antibody titers with comparable levels among the different groups (B-H1, 5 × 10^2^; B-H3, 1 × 10^6^; and B-Yam, 5 × 10^5^ PFU/mouse respectively). A group receiving phosphate-buffered saline (PBS) was used as a control. Mice of different ages, including young adults (3 months postpriming), mature adults (6 to 9 months postpriming), and early senescent adults (12 months postpriming), were challenged with different doses of H5N1 virus (10^1^ to 10^5^ PFU/mouse), and the 50% lethal dose (LD_50_) in every group was determined (54). At 3, 6, 9, and 12 months after the priming infection, the 50% lethal dose (LD_50_) of H5N1 virus in every group of primed mice was determined (10^1^ to 10^5^ PFU/mouse). Blood samples were collected before challenge at the indicated time points and 21 days postchallenge with H5N1. For ELISAs, serum samples were analyzed individually for every mouse, and area under the curve (AUC) values were reported. For ADCC reporter assays, samples from every individual group and every individual time point were pooled for each dose of the H5N1 challenge virus (maximum of three mice per group). Analysis was performed in duplicates, and AUC values were reported.

### ELISA.

Antibodies in mouse sera were measured as previously described (22). Briefly, ultrahigh binding polystyrene 96-well plates (Immulon 4HBX; Thermo Fisher Scientific) were coated with 100 μl/well of recombinant protein in PBS (pH 7.4) (Gibco) at a concentration of 2 μg/ml and incubated at 4°C overnight. The plates were washed three times with PBS containing 0.1% Tween 20 (PBS-T) with an automated plate washer system (AquaMax 2000; Molecular Devices). Unspecific binding was prevented by blocking with a solution (220 μl/well) of PBS-T, 3% goat serum (Gibco), and 0.5% nonfat dry milk (AmericanBio) for 1 to 2 h. Serum samples from every mouse were serially diluted (threefold) starting from a 1:100 dilution. Samples were added to the plates (100 μl/well), which were incubated at room temperature (RT) for 2 h and then washed three times. The secondary anti-mouse IgG H&L peroxidase-conjugated antibody (50 μl/well; Rockland) was added at a 1:3,000 dilution for 1 h at RT, the plates were washed four times, and the substrate *o*-phenylenediamine dihydrochloride (Sigmafast OPD; Sigma-Aldrich) was added (100 μl/well). After a 30-min incubation, 50 μl/well of a 3 M HCl solution (Thermo Fisher Scientific) was added to stop the reaction. Optical density (OD) was measured (490 nm) using a microplate reader (Synergy H1; Biotek). Analysis was performed using Prism 7 software (GraphPad), and values were reported as area under the curve (AUC).

### Antibody-dependent cellular cytotoxicity reporter assay.

Antibody effector functions were assessed using a commercial ADCC reporter assay kit (Promega) as described by the manufacturer’s instructions. For this, MDCK cells were seeded into 96-well white flat-bottom plates (Costar) at a density of 3 × 10^4^cells/well. The plates were incubated overnight at 37°C with 5% CO_2_. The cells were infected with A/Vietnam/1203/04 H5N1 (A/PR/8/34 reassortant as described above) virus at a multiplicity of infection (MOI) of 2, and infection was left to proceed for 16 to 18 h. Serial dilutions (threefold) of serum samples were performed in assay buffer consisting of RPMI 1640 medium supplemented with 0.5% low IgG FBS (Promega) starting from an initial dilution of 1:50. The supernatant of H5N1-infected MDCK cells was removed, and 25 μl/well of assay buffer and 25 μl/well of serum dilutions were added. Effector cells were thawed, washed with RPMI 1640 medium, and resuspended in assay buffer to a density of 3 × 10^6^ cells/ml. Twenty-five microliters of the suspension containing 7.5 × 10^4^ cells was added to each well. The plates were incubated at 37°C with 5% CO_2_ for 6 h, followed by the addition of 75 μl/well of Bio-Glo luciferase assay reagent (Promega). Luminescence was measured using a microplate reader (Synergy H1; Biotek, USA). Prism 7 software (GraphPad, USA) was used for data analysis, and values were reported as AUC.

### Statistical analysis.

Differences between prechallenge and postchallenge responses among different groups were determined using a regular two-way analysis of variance (ANOVA) with Dunnett multiple-comparison test. Analyses were performed using Prism 7 (GraphPad, USA). All adjusted *P* values of <0.05 were considered statistically significant with a confidence interval of 95%.
