# Supplementary material for: H1 Hemagglutinin Priming Provides Long-Lasting Heterosubtypic Immunity against H5N1 Challenge in the Mouse Model
Source: mBio. 2020 Dec 15;11(6):e02090-20. doi: 10.1128/mBio.02090-20 (PMC7773984; doi:10.1128/mBio.02090-20)
Supplement: TABLE S1 [file mBio.02090-20-st001.pdf]

Supplementary Table 1

Differential lethality of H5N1 in mice primed with heterosubtypic strains

**Priming group**	**Influenza H5N1 lethal dose 50% (LD_{50})**			
	**Day 90**	**Day 180**	**Day 270**	**Day 360**
B-H1	$5.6x10^3$	$3.2x10^4$	$1.8x10^4$	$3.2x10^4$
B-H3	$3.2x10^2$	$3.2x10^3$	$3.2x10^3$	$3.2x10^3$
WT	$5.6x10^2$	$3.2x10^3$	$1.8x10^3$	$3.2x10^3$
PBS	$3.2x10^2$	$3.2x10^3$	1.810^3	$3.2x10^3$
