# Supplementary material for: H1 Hemagglutinin Priming Provides Long-Lasting Heterosubtypic Immunity against H5N1 Challenge in the Mouse Model
Source: mBio. 2020 Dec 15;11(6):e02090-20. doi: 10.1128/mBio.02090-20 (PMC7773984; doi:10.1128/mBio.02090-20)
Supplement: FIG S1 [file mBio.02090-20-sf001.pdf]

Supplementary Figure 1

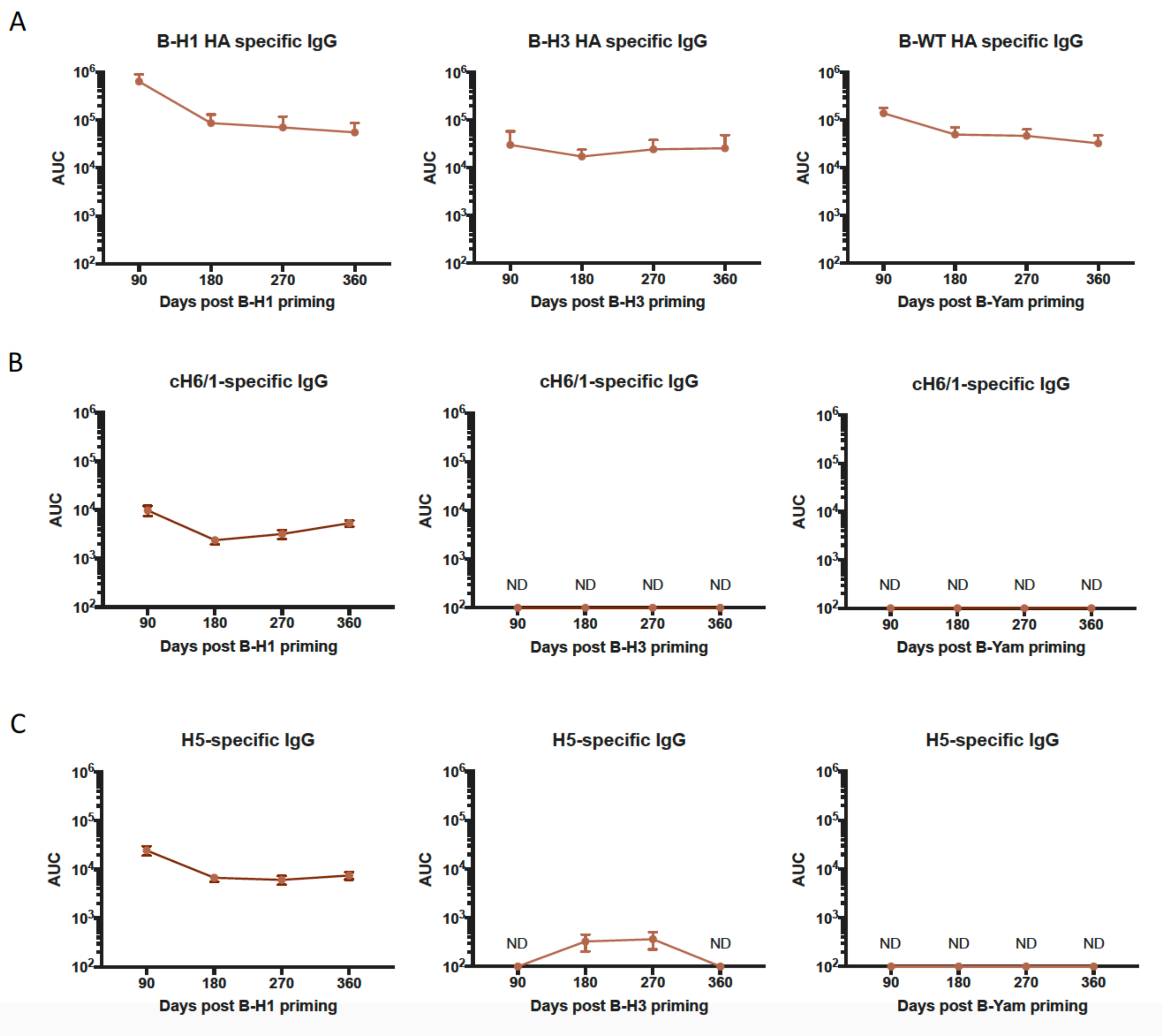

Supplementary figure 1. Pre-challenge IgG levels against the specific HA from the corresponding priming strains, H5 and cH6/1. Mice were primed with B-H1, B-H3, B/Yamagata/16/88 viruses or PBS treated (n=60/group). 90, 180, 270 or 360 days post-priming,

sera from all the mice were collected. Specific IgG against the corresponding HA from the priming strains (H1 for B-H1, H3 for B-H3, and B-HA for B-WT) (A), the chimeric HA cH6/1 (B), or H5 from A/Vietnam/1203/04 virus (C) was measured. Antibody levels are expressed as area under the curve (AUC). Mean plus SEM is plotted for each group.
